# An uncommon ECG manifestation of normal pacemaker function

**DOI:** 10.1002/joa3.12258

**Published:** 2019-11-12

**Authors:** Sharath K. Kumar, Christopher D. Morgan, Michael Jordan‐Watt, Sheldon M. Singh

**Affiliations:** ^1^ Sunnybrook Health Sciences Center University of Toronto Toronto ON Canada; ^2^ Biotronik Canada Inc. Toronto ON Canada

**Keywords:** artifact, closed loop stimulation, electrocardiogram, electromagnetic interference, pacemaker

## Abstract

A presumed abnormal electrocardiogram (ECG) was obtained from an asymptomatic patient with a pacemaker. Systematic evaluation of the ECG revealed that the artifact was due to a physiological sensor in the pacemaker which was displayed when the enhanced pacemaker detection features on the ECG machine was activated. The article discusses the possible causes and an approach to similar artifacts.

## Graphical abstract

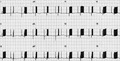

## 1 INTRODUCTION

An asymptomatic patient with a permanent pacemaker was reported to have an abnormal ECG with a characteristic artifact.

## 2 CASE REPORT

**Figure 1:**
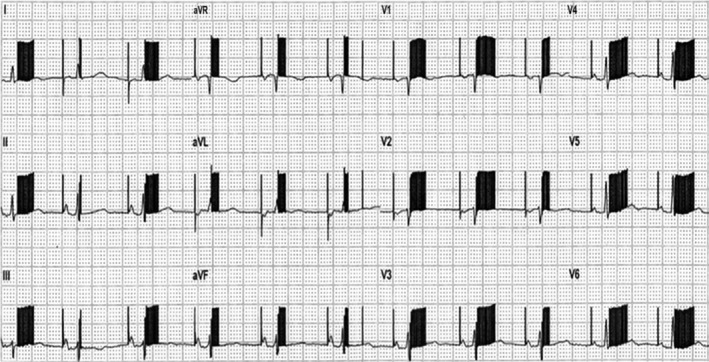
ECG with pacemaker enhancement algorithm turned on during the use of the closed loop stimulation algorithm

This presumed abnormal ECG (Figure 1) was obtained from an asymptomatic patient with a permanent pacemaker.

Electro‐magnetic interference from external devices including neuro‐muscular stimulators is an unlikely explanation for the ECG findings given the consistent linking of the artifact to each QRS complex. Tremor artifacts are continuous, low amplitude and rhythmic and thus not the cause of the observed artifact. While cardiac myoplasty stimulation may result in artifact associated with each QRS complex, one would not expect prolonged consistent high frequency signals with each QRS, nor would one expect a normal QRS morphology in this rare situation.^1^

Normal pacemaker function may produce ECG artifact related to physiologic sensors utilizing sub‐threshold pacing to measure intra‐cardiac and intra‐thoracic impedance. One such example is the closed loop stimulation sensor unique to Biotronik (Biotronik GmbH) pacemakers. This algorithm performs eight biphasic impedance measurements (obtained with 17 sub‐threshold paced beats) 50‐300 milliseconds after a ventricular event to determine intracavitary impedance to guide physiologic rate response.^2^ For these unipolar impulses to be recognized on the surface ECG, more frequent sampling is required to reveal these impulses. Sub‐threshold pacing associated with this sensor may be made more prominent when enhanced pacemaker detection features (which alter filtering and processing of ECG signals) are activated in ECG machines.^3^ Since the detection algorithms are still imperfect, there may be variability in the number of spikes detected per beat.^4^ This could account for variability in the spikes observed on the surface ECG.

Further history confirmed the presence of a Biotronik pacemaker with activation of the closed looped stimulation algorithm. Additionally, interrogation of the ECG machine revealed utilization of the enhanced pacemaker detection feature during ECG acquisition. A repeat ECG after deactivation of this feature resulted in the absence of artifact.

## 3 CONCLUSIONS

This case highlights the value of a systematic approach to interpreting ECG artifacts and knowledge of the influence of enhanced pacemaker detection features on ECGs.

## CONFLICT OF INTERESTS

Mr Jordan‐Watt is an employee of Biotronik, Canada. Drs. Kumar, Morgan and Singh have no relevant conflict of inflict to disclose.
